# Doctors in Camera

**Published:** 1897-07-31

**Authors:** 


### Doctors in Camera.

The annual meeting of the British Medical Association was opened on Tuesday at Exeter Hall. The proceedings were, perhaps, hardly worthy of so important a body; but that is a small matter. Those who are accustomed to attend these meetings have long ceased to expect that they should be conducted with especial dignity, and on this occasion the traditions of the Association were fairly maintained. As one member was heard to say, on his way to lunch, "It was rare fun."

Fun though it was, however, to some, to those who have the interests of the Association at heart there was much to cause disquietude, partly in the nature of the business which came before the meeting, partly in the manner in which it was dealt with. It always seems to us a fatuous proceeding to exclude reporters from a public meeting which is open to an immense number of persons, everyone of whom is absolutely at liberty to tell as many people as he likes every word of the discussion.

Seeing, then, that by no pretence could it be maintained that this was a private meeting, and that even if the Chairman had chosen to say that the proceedings were confidential, no one would have been in the slightest degree bound to accept his ruling, the exclusion of reporters when a certain point in the report was reached merely served to excite curiosity, and to suggest that something was coming of which the Council were ashamed. In any case, while the reporters were absent the meeting managed at the instigation of the Council to commit a great injustice, and to drag the Association which it represented down to the level of the lowest and most unreasoning trades unionism.

We have already on more than one occasion drawn attention to the ill-advised proceedings of the Council in expelling certain members, not for breaches of generally recognised ethical rules, but merely because they had acted in opposition to local professional opinion. It might have been expected that when the resolution of the Council expelling the two gentlemen referred to came up for confirmation at the general meeting, some sense of fairness would have been exhibited. But no. Not a particle of evidence of wrong conduct was given; not a suggestion was made of their having done anything more than accept office at the Adelaide Hospital at a time when the whole staff had chosen to resign in consequence of the action of the Government; not a single word was uttered to show that the resignation of the staff was justifiable, or that the South Australian Government was wrong in what it had done to replace them. Yet the meeting blindly accepted the ruling of the Council, and confirmed the act of injustice which they had perpetrated. To the honour of Surgeon-Major Ince, it should be mentioned that he pointed out to this hostile meeting the extreme gravity of the issue raised. It should also be mentioned that, notwithstanding efforts to repress the information, the fact was elicited that the expelled members had gone out at the invitation of a member of the staff of one of our largest London hospitals. But the meeting would have none of it. It was officially put before them that they had simply to deal with the plain fact that when no one could be found in Adelaide to take the posts vacated by the staff of the Adelaide Hospital, these two gentlemen were willing to go out to South Australia and accept office, and on this issue, amid calls of "blacklegs," their expulsion was confirmed.

The British Medical Association must take the conquences of its own folly. So far as its action at this meeting can be taken as a precedent, it means that any member who makes himself objectionable to his neighbours may be expelled, and that his expulsion may be confirmed at a general meeting without the production of a word of proof.

If the British Medical Association were openly and professedly a trades union, people would know what to expect when they joined its ranks. Curiously enough, however, it poses as a scientific body. But even in a trades union people would not be expelled unless they had broken some rule definitely laid down and properly promulgated, whereas in this case there was no pretence that such a rule had been broken, or had indeed even existed. No member is safe in an Association which makes up its ethics as it goes along, and distributes its punishments in deference to popular clamour.

